# Absolute Reliability of Gait Parameters Acquired With Markerless Motion Capture in Living Domains

**DOI:** 10.3389/fnhum.2022.867474

**Published:** 2022-06-16

**Authors:** Sherveen Riazati, Theresa E. McGuirk, Elliott S. Perry, Wandasun B. Sihanath, Carolynn Patten

**Affiliations:** ^1^Biomechanics, Rehabilitation, and Integrative Neuroscience Lab, Department of Physical Medicine and Rehabilitation, School of Medicine, University of California, Davis, Sacramento, CA, United States; ^2^UC Davis Healthy Aging in a Digital World Initiative, a UC Davis “Big Idea”, Sacramento, CA, United States; ^3^Center for Neuroengineering and Medicine, University of California, Davis, Davis, CA, United States; ^4^VA Northern California Health Care System, Martinez, CA, United States

**Keywords:** measurement error, repeatability, digital biomarkers, kinematics, rehabilitation outcomes, gait, markerless motion capture, spatiotemporal gait parameters

## Abstract

**Purpose**: To examine the between-day absolute reliability of gait parameters acquired with Theia3D markerless motion capture for use in biomechanical and clinical settings.

**Methods**: Twenty-one (7 M,14 F) participants aged between 18 and 73 years were recruited in community locations to perform two walking tasks: self-selected and fastest-comfortable walking speed. Participants walked along a designated walkway on two separate days.Joint angle kinematics for the hip, knee, and ankle, for all planes of motion, and spatiotemporal parameters were extracted to determine absolute reliability between-days. For kinematics, absolute reliability was examined using: full curve analysis [root mean square difference (RMSD)] and discrete point analysis at defined gait events using standard error of measurement (SEM). The absolute reliability of spatiotemporal parameters was also examined using SEM and SEM%.

**Results**: Markerless motion capture produced low measurement error for kinematic full curve analysis with RMSDs ranging between 0.96° and 3.71° across all joints and planes for both walking tasks. Similarly, discrete point analysis within the gait cycle produced SEM values ranging between 0.91° and 3.25° for both sagittal and frontal plane angles of the hip, knee, and ankle. The highest measurement errors were observed in the transverse plane, with SEM >5° for ankle and knee range of motion. For the majority of spatiotemporal parameters, markerless motion capture produced low SEM values and SEM% below 10%.

**Conclusion**: Markerless motion capture using Theia3D offers reliable gait analysis suitable for biomechanical and clinical use.

## Introduction

Walking represents an individual’s capacity for independence, autonomy, and self-efficacy making it an important benchmark of human health. Biomechanical gait analysis has been used extensively for evaluating human movement with findings applied in clinical populations to identify changes that occur in neuropathologies (Chen et al., 2005a; Albani et al., 2014). Gait is such a fundamental manifestation of human health that White et al. (2013) proposed gait speed, a spatiotemporal parameter (STP), as the sixth vital sign. More recently, various gait parameters (e.g., reduced hip flexion range of motion, increased stride time variability, swing time, step width) have been proposed as biomarkers for disease identification, progression, and rehabilitation outcomes in neuropathologies and cognitive disorders (Chen et al., 2005a, 2011; Jonkers et al., 2009; McDonald et al., 2013; Albani et al., 2014; Mancini et al., 2015; Valkanova et al., 2018; De Cock et al., 2019).

Joint kinematics, generated through biomechanical analyses, can be assessed over the entire time series of a gait cycle through curve analysis (Edwards et al., 2017) which offers the ability to characterize the pattern of excursion across the entire gait cycle (Fellin et al., 2010). For clinical use, however, gait analysis typically focuses on key determinants of gait such as the ankle, knee, and hip joint angles at specific events within the gait cycle, and may evaluate joint angles at initial contact, maximum angles in stance phase, and range of motion (RoM) across the entire gait cycle (El-Tamawy et al., 2012; Bonnyaud et al., 2015). Each of these variables has been used to explore movement behaviors in individuals with pathological gait (Chen et al., 2005a, b; Chen and Patten, 2008; Tenniglo et al., 2018; Penko et al., 2020). Additionally, STPs such as gait speed, cadence, stance, swing, and stride time, are used to describe gait dysfunction (Chen et al., 2005a, b), while STP variability has been used to characterize age-related and cognitive decline (Bahureska et al., 2017; Ceïde et al., 2018; De Cock et al., 2019).

Gait analysis is typically measured in a dedicated laboratory requiring specialized equipment and expertise. Laboratory three-dimensional (3D) motion capture systems rely on researcher expertise to perform data collection where required anthropometric measures such as stature, leg length, knee, and ankle breadth are required (Sun et al., 2020; Moreira et al., 2021; Reznick et al., 2021). Marker placement on specific anatomical landmarks is a critical skill that influences the accuracy of calculations derived from the collected data. Indeed, marker placement has been identified as a key source of error in motion analysis (Ferber et al., 2002; Noehren et al., 2010). For example, a marker misplacement of 10 mm on the ankle or knee can cause an error up to 7° (Osis et al., 2016). An additional challenge is for securing markers to reduce noise resulting from skin and tissue artifacts (Akbarshahi et al., 2010; Tsai et al., 2011; Osis et al., 2016).

Wearable inertial measurement units (IMUs) address some of these challenges inherent to 3D motion capture and also offer potential for cost-effective portable clinical gait analysis (Wu et al., 2021; McDevitt et al., 2022). Wearable devices such as the APDM Opal IMU-based system have been promoted for use in clinical populations to monitor gait and mobility in individuals with neurodegenerative conditions such as Parkinson’s disease and have recently been advanced as digital biomarker endpoints for clinical trials (Mancini et al., 2015; Mancini and Horak, 2016). IMU development has seen much progress and is under rapid expansion with offering the potential for accurate detection of kinematics (Al Borno et al., 2022).

In addition to IMUs, markerless motion capture (MLMC) systems have been under development by multiple investigators for purposes ranging from surveillance to motion analysis (Mündermann et al., 2006a, 2007). Several authors have worked on progressing the technology for clinical use (Mündermann et al., 2006a, b; Sandau et al., 2014; Kanko et al., 2021a, b; Lonini et al., 2022). Contributions from the fields such as machine learning have made critical advances to the accuracy and feasibility of this approach (Mündermann et al., 2007; Corazza et al., 2010). Corazza et al. (2010) introduced an automatic generation of subject-specific models that produced a comparable error in joint identification to marker placement errors for 3D motion capture. Implementation of MLMC comes with an underlying premise for robust detection and quantification of gait (Kanko et al., 2021a, b, c). Furthermore, MLMC technology improves data collection efficiency by eliminating the need for marker placement while providing accurate, high-resolution data (Sandau et al., 2014; Verlekar et al., 2019). Importantly, while IMUs may reduce some burden associated with data collection, they still require direct contact with the participant and accurate placement of sensors on body segments (Mancini and Horak, 2016; Al Borno et al., 2022). A distinct advantage of MLMC is the ability to capture data without the need for a dedicated laboratory or requirements for specialized attire, including footwear. Freedom from these requirements greatly improves the accessibility of gait analysis to clinical and community settings while simultaneously lowering the experimental burden on participants.

Theia3D, a machine learning algorithm-based MLMC software, has been recently developed with an aim for clinical use (Kanko et al., 2021a, b, c). This algorithm uses current biomechanical standards (i.e., inverse kinematics and rigid body tracking) to estimate the three-dimensional pose of the body segments. The Theia3D algorithm has been carefully examined by the developers demonstrating comparable outcomes to kinematics acquired with the putative gold standard, marker-based 3D motion capture (Kanko et al., 2021a). Kanko et al. (2021a) examined lower extremity joint motions during gait and found that data acquired using MLMC are comparable to traditional maker-based methods in sagittal, frontal, and transverse planes. Subsequently, Kanko et al. (2021b) reported repeatable inter-session data using MLMC by evaluating the variability of segmental kinematics captured across the full gait cycle (i.e., curves). Their analysis, however, did not provide a between-day measurement error of discrete points associated with Theia3D.

Assessment of measurement error is necessary for discriminating between healthy and abnormal gait while avoiding over-interpretation of small differences such as those intrinsic to human motor behavior or technical attributes of instrumentation (Schwartz et al., 2004; McGinley et al., 2009). Absolute reliability can provide clinicians a value of the magnitude of measurement error associated with the equipment and an index of expected trial-to-trial noise in the data (Weir, 2005). For kinematics, measurement errors typically reported from marker-based methods range from 2 to 5°, while errors greater than 5° are cause for concern (McGinley et al., 2009).

While the developers have demonstrated data acquired with MLMC that may be comparable to marker-based motion capture with acceptable inter-session variation (Kanko et al., 2021a, c), the absolute reliability of kinematic data acquired with Theia3D has not been reported. The aims of this study are to investigate (i) test-retest (between days) measurement error of lower extremity kinematics—(a) over the entire joint curve; and (b) at specific points within the gait cycle commonly used to evaluate gait; and (ii) spatiotemporal parameters of gait acquired using MLMC. The data captured during this study were collected in community and clinic-adjacent settings to establish the feasibility of using MLMC for gait analysis as a clinical outcome for the assessment of impairment and intervention effects in persons with neuropathological conditions.

## Methods

### Participants

Following a review of literature (McGinley et al., 2009), we determined that 20 participants were necessary to establish reliability. A total of 21 individuals meeting the following broad criteria: (i) ability to follow three-step commands; (ii) ability to ambulate 15 meters, independently; and (iii) ability to provide written informed consent, participated in two sessions of gait assessment conducted in community and clinically-adjacent settings (detailed in the companion article, McGuirk et al., 2022). Participant characteristics and demographics are presented in Table 1. Participants were recruited in real-time, thus were given no prior instruction regarding clothing or footwear. Participants provided electronically written informed consent and completed health history and demographics questionnaires hosted on the Research Electronic Data Capture (REDCap) infrastructure (Harris et al., 2009, 2019). All procedures were approved by the University of California, Davis Institutional Review Board (#1386142) and conducted according to the Declaration of Helsinki.

**Table 1:** Participant characteristics represented as mean ± standard deviation.

Participants (n)	21
Male	7
Female	14
Age (years)	37.8 ± 18.8
Height (m)	1.7 ± 0.1
Mass (kg)	70.8 ± 11.0
Leg Length (m)	
Right	0.84 ± 0.1
Left	0.84 ± 0.1
Race/Ethnicity (n)	
Asian, not Hispanic, or Latino	3
More than one race, not Hispanic or Latino	2
White, Hispanic, or Latino	2
White, not Hispanic, or Latino	14

### Data Capture

**Figure 1:**
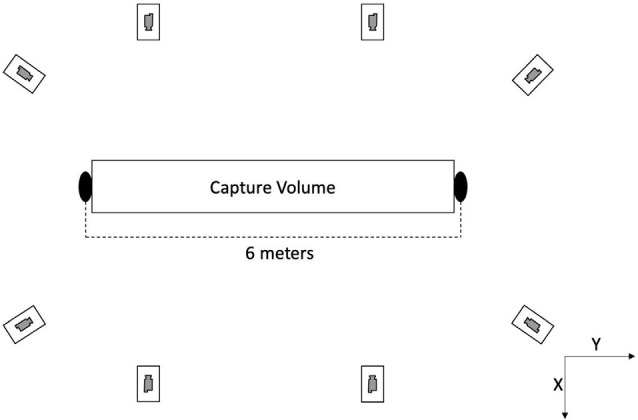
Illustrating data capture experimental setup.

Gait analysis was performed at three University of California, Davis locations. Nineteen (of 21) participants were studied in the same location. All participants performed two sessions separated by 10 ± 12 days (range, 1–52 days). The same camera setup was deployed at all locations (see Figure 1). Eight video cameras (Basler ace acA1300-75gc GigE, Ahrensburg, Germany) were arranged to produce a capture volume approximately six meters long, four meters wide, and two meters high. Four cameras were positioned along each side of the walkway with cameras nearest the ends of the walkway shifted 0.5 m to the center. The cameras were calibrated during set up at each location and recalibrated between each data collection session. Brightly colored sports cones were placed 10 m apart at the far ends of the walkway to mark the start and finish targets. Synchronized videos were collected at 60 Hz using AccuPower 4.0 software (v1.3.6.1978 Treadmetrix, Park City, Utah, USA).

### Procedure

Participants performed two walking tasks: self-selected walking speed (SSWS) and fastest comfortable walking speed (FCWS). For the SSWS task, participants were instructed to walk normally at their usual pace. For FCWS, participants were instructed to imagine seeing oncoming traffic while being in a crosswalk and navigating to safety without running or jogging. Participants performed approximately six to eight passes for each task. These procedures were repeated in the second session.

### Data Analysis

Video data were processed using Theia3D (v2021.2.0.1675, Theia Markerless Inc., Kingston, ON, Canada) to obtain three-dimensional (3D) subject pose estimates of limb segments. The resulting 4 × 4 pose estimates for each body segment were exported to Visual3D Professional (v2021.06.02, C-Motion, Inc., Germantown, MD, USA) for further analysis. The gait events, initial contact (IC), and toe-off, were determined using methods described by Zeni et al. (2008), with quality checks performed in Visual3D. Lower limb joint kinematics for the hip, knee, and ankle were calculated for the sagittal, frontal, and transverse planes (see Table 2). Variables of interest at defined points in the gait cycle and spatiotemporal parameters were calculated and analyzed in MATLAB (v2020a, Mathworks, Natick, MA, USA; see Table 3). Data were analyzed from the participant’s self-identified dominant leg. For each condition, the number of strides was matched between the two sessions.

**Table 2:** Gait event descriptions and abbreviations.

**Gait Event**	**Description**
ST1	Stance phase I, ipsilateral initial contact to mid-stance, representing the first half of the stance phase	
ST2	Stance phase II, mid-stance to ipsilateral toe-off, representing the second half of the stance phase	
ST/SW	Transition from stance-to-swing phase	
RoM	The entire range of motion over the full gait cycle, presented for hip, knee, and ankle joints in sagittal, frontal, and transverse planes
MaxFlex_Stance_	Maximum flexion angle (sagittal plane) achieved by the hip or knee joint during the stance phase
MaxFlex_Swing_	Maximum flexion angle (sagittal plane) achieved by the hip or knee joint during the swing phase
MaxFlex_ST1_	Maximum flexion angle (sagittal plane) achieved by the knee joint during stance phase I
MaxExt_Stance_	Maximum extension angle (sagittal plane) achieved by the hip or knee joint during the stance phase
MaxExt_Swing_	Maximum extension angle (sagittal plane) achieved by the hip or knee joint during swing phase
MaxDorsiflexion_ST2_	Maximum dorsiflexion angle (sagittal plane) achieved by the ankle joint during the stance phase II
MaxPlantarflexion_ST/SW_	Maximum plantarflexion angle (sagittal plane) of the ankle joint at stance-to-swing transition
MaxAdd_DLS1_	Maximum adduction angle (frontal plane) achieved by the hip joint during double limb support phase I
MaxAbd_Swing_	Maximum abduction angle (frontal plane) achieved by the hip joint during the swing phase
MaxVarus_Swing_	Maximum varus angle (frontal plane) achieved by the knee joint in the swing phase
MaxInv_Swing_	Maximum inversion angle (frontal plane) achieved by the ankle joint during the swing phase
MaxEv_ST1_	Maximum eversion angle (frontal plane) achieved by the ankle joint during the stance phase I
MaxIntRot_ST1_	Maximum internal rotation (transverse plane) achieved by the hip or ankle joint during the stance phase I
MaxExtRot_ST2_	Maximum external rotation (transverse plane) achieved by the hip or ankle joint during the stance phase II
MaxExtRot_Swing_	Maximum external rotation (transverse plane) achieved by the hip or ankle joint during the swing phase
AnkleInitialContact	Ankle joint angle (sagittal or frontal plane) at initial contact
KneeMidStance	Knee joint angle (frontal or transverse plane) at mid-stance	
KneeMidSwing	Knee joint angle (frontal or transverse plane) at mid-swing	

**Table 3:** Sagittal plane variables—Self-Selected Walking Speed: mean ± standard deviation (std) for Session 1 and Session 2, and Standard Error of Measurement (SEM).

		Session 1	Session 2	
*Sagittal*		Mean ± std	Mean ± std	SEM
Hip_(degrees)_				
	RoM	46.2 ± 5.4	47.6 ± 4.5	2.16
	MaxFlex_Stance_	26.2 ± 3.6	26.7 ± 3.5	1.81
	MaxFlex_Swing_	26.1 ± 3.1	26.9 ± 2.8	1.50
	MaxExt_Stance_	−18.9 ± 4.2	−19.7 ± 2.9	1.94
				
Knee_(degrees)_				
	RoM	65.1 ± 4.1	65.8 ± 3.9	1.96
	MaxFlex_ST1_	16.8 ± 6.7	17.5 ± 6.9	2.36
	MaxFlex_Swing_	63.8 ± 3.7	64.9 ± 3.9	1.66
	MaxExt_Stance_	2.7 ± 4.2	
2.8 ± 3.4	1.78
				
Ankle_(degrees)_				
	RoM	39.3 ± 4.6	41.7 ± 5.7	2.47
	InitialContact	−4.6 ± 3.5	−5.2 ± 4.3	2.84
	MaxDorsiflexion_ST2_	10.1 ± 2.8	10.6 ± 4.2	1.82
	MaxPlantarflexion_ST/SW_	−28.9 ± 4.6	−31.0 ± 4.4	2.42

### Statistical Analysis

Error examination was performed using the approach described by Kanko et al. (2021a) where root-mean-square differences (RMSD) were calculated to evaluate between-day differences in the full curves of all three joints and planes of motion. For each session, the average RMSD was computed using the ensemble average of the participant’s within-session data. Absolute reliability was examined using standard error of measurement (SEM) of discrete points within the gait cycle and spatiotemporal parameters (Weir, 2005). Standard error of measurement represents a combination of random and systematic error and quantifies the precision of the equipment. Additionally, the SEM provides a value of error in the same units as the measurement (Weir, 2005). Measurement errors between 2° and 5° are to be considered acceptable (McGinley et al., 2009). The percentage of SEM expressed from the mean (SEM%) was derived for all spatiotemporal parameters. All statistical analysis was performed *via* custom-written MATLAB scripts and SPSSv27 (SPSS Inc., Chicago, IL, USA).

## Results

**Figure 2:**
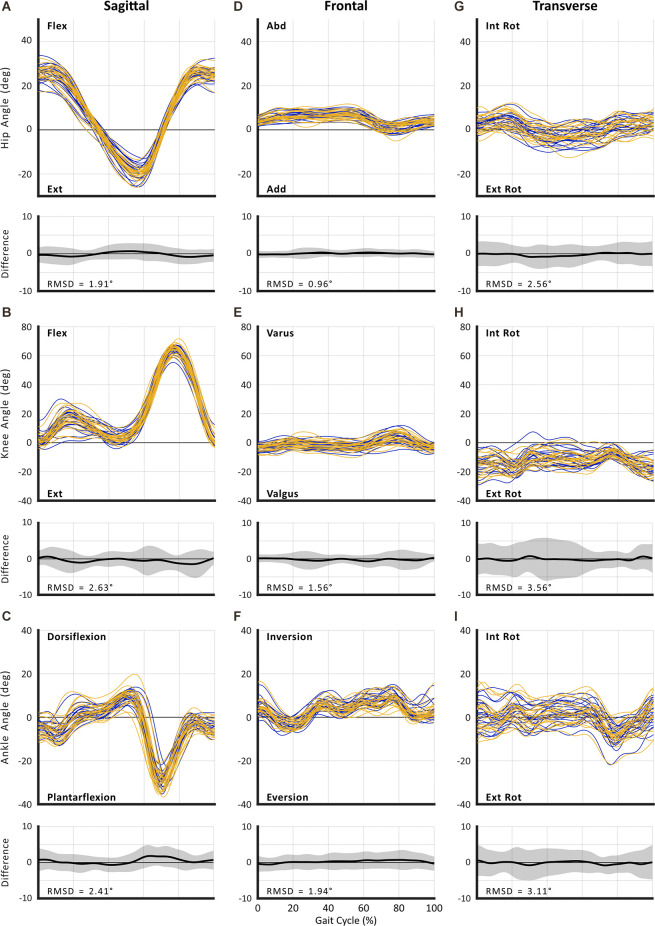
Between-session reproducibility of full curve analysis for subject-average joint angles during self-selected walking speed (SSWS). Data illustrate joint angles for hip (row 1), knee (row 2), and ankle (row 3) in the sagittal **(A–C)**, frontal **(D–F)**, and transverse planes **(G–I)**. Each curve represents the ensemble average of each individual’s trials for Session 1 (blue lines) and Session 2 (gold lines). The average RMS difference across all subjects is shown below the respective joint angle plot; across all joints and planes of motion, the largest RMSD was 3.71°.

**Figure 3:**
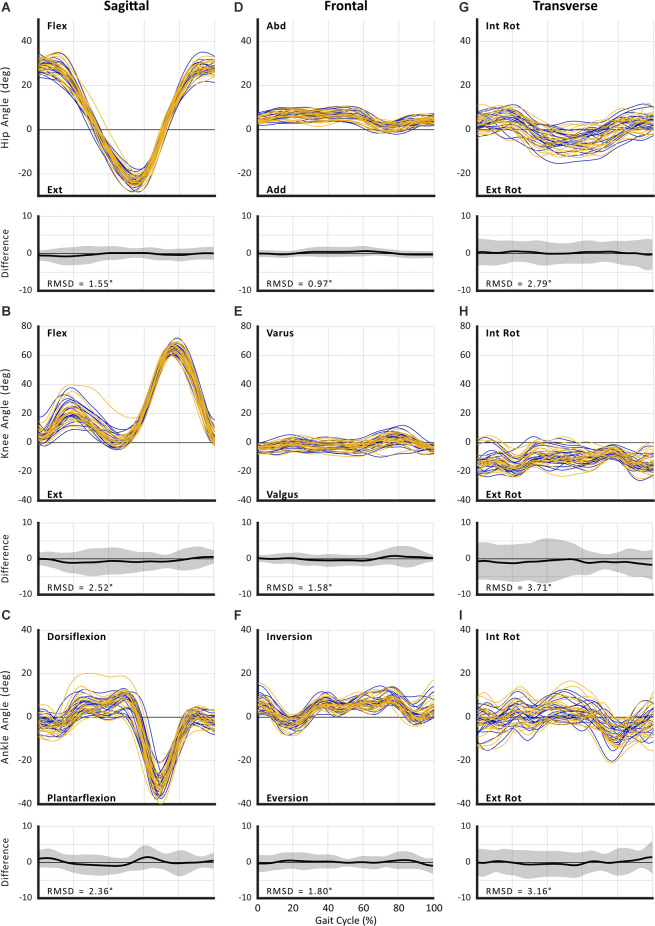
Between-session reproducibility of full curve analysis for subject-average joint angle during fastest comfortable walking speed (FCWS). Data illustrate joint angles for hip (row 1), knee (row 2), and ankle (row 3) in the sagittal **(A–C)**, frontal **(D–F)**, and transverse planes **(G–I)**. Each curve represents the ensemble average of each individual’s trials for Session 1 (blue lines) and Session 2 (gold lines). The average RMS difference across all subjects is shown below the respective joint angle plot; across all joints and planes of motion, the largest RMSD was 3.56°.

The RMSDs from full curve analysis of kinematics for both SSWS and FCWS are reported in Figures 2 and 3. Absolute reliability expressed as SEM and RMSD was <5° for the majority of variables, thus deemed acceptable (McGinley et al., 2009). Results of discrete point kinematics and spatiotemporal variables expressed as SEM are presented in Tables s 3–9.

**Table 4:** Frontal plane variables—Self-Selected Walking Speed: mean ± standard deviation (std) for Session 1 and Session 2, and Standard Error of Measurement (SEM).

		Session 1	Session 2	
*Frontal*		Mean ± std	Mean ± std	SEM
Hip_(degrees)_				
	RoM	7.4 ± 2.6	7.7 ± 3.2	1.05
	MaxAdd_DLS1_	6.4 ± 2.2	6.7 ± 2.3	1.03
	MaxAbd_Swing_	0.45 ± 2.1	0.23 ± 2.6	0.91
Knee_(degrees)_				
	RoM	9.8 ± 4.0	10.6 ± 4.0	2.53
	MidStance	−2.0 ± 2.7	−1.4 ± 3.4	1.61
	MaxVarus_Swing_	4.74 ± 4.2	5.4 ± 4.3	2.82
Ankle_(degrees)_				
	RoM	16.2 ± 4.4	16.0 ± 4.1	3.01
	Initial Contact	4.3 ± 4.3	4.6 ± 4.1	2.18
	MaxInv_Swing_	10.9 ± 3.6	10.3 ± 4.2	2.59
	MaxEv_ST1_	−4.1 ± 3.2	−4.3 ± 3.5	2.32
	MaxExt_Swing_	−0.18 ± 3.3	−0.86 ± 3.1	2.50

**Table 5:** Transverse plane variables—Self-Selected Walking Speed: mean ± standard deviation (std) for Session 1 and Session 2, and Standard Error of Measurement (SEM).

		Session 1	Session 2	
*Transverse*		Mean ± std	Mean ± std	SEM
Hip_(degrees)_				
	RoM	12.7 ± 3.7	12.6 ± 4.3	2.80
	MaxIntRot_ST1_	6.1 ± 3.4	6.4 ± 4.3	2.96
	MaxExtRot_ST2_	−5.2 ± 4.2	−4.2 ± 4.1	2.81
Knee_(degrees)_				
	RoM	19.2 ± 5.7	19.4 ± 6.3	4.69
	MidStance	−9.4 ± 7.2	−10.9 ± 6.7	4.11
	MidSwing	−10.9 ± 4.5	−11.0 ± 5.0	3.39
Ankle_(degrees)_				
	RoM	20.2 ± 6.6	21.0 ± 6.4	4.95
	MaxIntRot_ST1_	5.8 ± 5.7	6.4 ± 6.5	3.71
	MaxExtRot_Swing_	−11.3 ± 5.9	−10.5 ± 6.2	3.37

**Table 6:** Sagittal plane variables—Fastest Comfortable Walking Speed: mean ± standard deviation (std) for Session 1 and Session 2, and Standard Error of Measurement (SEM).

		Session 1	Session 2	
*Sagittal*		Mean ± std	Mean ± std	SEM
Hip_(degrees)_				
	RoM	53.3 ± 5.0	54.0 ± 4.4	1.82
	MaxFlex_Stance_	29.9 ± 3.1	30.3 ± 3.0	1.53
	MaxFlex_Swing_	28.8 ± 3.0	28.6 ± 2.8	1.48
	MaxExt_Stance_	−22.9 ± 3.1	−23.4 ± 2.6	1.40
Knee_(degrees)_				
	RoM	65.5 ± 5.0	65.9 ± 4.5	2.38
	MaxFlex_ST1_	21.2 ± 7.7	22.6 ± 7.6	2.54
	MaxFlex_Swing_	65.1 ± 3.9	65.6 ± 4.3	2.09
	MaxExt_Stance_	0.94 ± 4.2	
1.4 ± 5.1	2.83
Ankle_(degrees)_				
	RoM	40.3 ± 5.5	41.5 ± 6.4	2.52
	Initial Contact	−2.4 ± 4.1	−3.2 ± 4.8	2.59
	MaxDorsiFlexion_ST2_	8.7 ± 3.6	
9.4 ± 4.8	1.97
	MaxPlantarflexion_ST/SW_	−31.2 ± 4.7	−31.6 ± 4.8	2.02

**Table 7:** Frontal plane variables—Fastest Comfortable Walking Speed: mean ± standard deviation (std) for Session 1 and Session 2, and Standard Error of Measurement (SEM).

		Session 1	Session 2	
*Frontal*		Mean ± std	Mean ± std	SEM
Hip_(degrees)_				
	RoM	7.3 ± 2.4	7.3 ± 2.2	0.95
	MaxAdd_DLS1_	6.5 ± 2.4	6.8 ± 2.2	1.34
	MaxAbd_Swing_	0.72 ± 1.9	0.55 ± 1.9	0.91
Knee_(degrees)_				
	RoM	9.6 ± 3.6	9.6 ± 3.5	2.33
	MidStance	−2.2 ± 2.8	−1.8 ± 3.1	1.10
	MaxVarus_Swing_	4.3 ± 4.5	3.7 ± 3.7	2.28
Ankle_(degrees)_				
	RoM	15.0 ± 4.2	15.4 ± 5.0	3.25
	InitialContact	5.4 ± 4.3	5.9 ± 4.2	2.25
	MaxInv_Swing_	10.4 ± 3.6	10.1 ± 3.7	2.34
	MaxEv_ST1_	−3.0 ± 3.3	−3.7 ± 4.1	2.79
	MaxExt_Swing_	−0.18 ± 3.8	−0.04 ± 3.8	2.69

**Table 8:** Transverse plane variables—Fastest Comfortable Walking Speed: mean ± standard deviation (std) for Session 1 and Session 2, and Standard Error of Measurement (SEM).

		Session 1	Session 2	
*Transverse*		Mean ± std	Mean ± std	SEM
Hip_(degrees)_				
	RoM	14.7 ± 4.1	14.9 ± 4.8	3.39
	MaxIntRot_ST1_	6.5 ± 3.9	6.5 ± 4.4	2.62
	Max Ext Rot _ST2_	−7.0 ± 4.4	−7.2 ± 4.8	2.52
Knee_(degrees)_				
	RoM	17.6 ± 5.1	18.4 ± 6.5	5.35
	MidStance	−9.6 ± 6.5	−9.1 ± 6.6	3.98
	MidSwing	−10.8 ± 4.5	−9.5 ± 4.9	3.73
Ankle_(degrees)_				
	RoM	19.5 ± 5.4	20.6 ± 6.6	5.50
	MaxIntRot_ST1_	4.5 ± 5.6	5.1 ± 5.7	3.34
	MaxExtRot_wing_	−11.4 ± 5.2	−11.9 ± 6.7	4.49

**Table 9:** Mean ± standard deviation (std) of the variables for both sessions (session 1 and session 2) along with Standard Error of Measurement (SEM), and percentage of Standard Error of Measurement expressed from the mean (SEM%) of Spatio-temporal variables during both self-selected walking speed (SSWS) and fastest comfortable walking speed (FCWS).

		Session 1	Session 2		
*Task*		Mean ± std	Mean ± std	SEM	SEM%
SSWS					
	Gait Speed (m.s^−1^)	1.35 ± 0.26	1.41 ± 0.26	0.07	5.3
	Gait Speed_LL (m.s^−1^)	1.59 ± 0.28	
1.68 ± 0.29	0.09	5.2
	Cadence (steps/min)	109.5 ± 10.65	111.95 ± 10.00	2.92	2.6
	Stride Time (sec)	1.11 ± 0.12	1.08 ± 0.10	0.04	3.8
	Stance Time (sec)	0.74 ± 0.10	0.72 ± 0.07	0.04	5.5
	Double Leg Support Time 1 (sec)	0.18 ± 0.04	
0.17 ± 0.03	0.02	10.5
	Double Leg Support Time 2 (sec)	0.18 ± 0.05	
0.17 ± 0.03	0.03	16.8
	Single Leg Support Time (sec)	0.38 ± 0.04	
0.37 ± 0.03	0.02	4.6
	Swing Time (sec)	0.38 ± 0.05	0.37 ± 0.03	0.03	7.0
	Stride Length (meters)	1.43 ± 0.19	1.47 ± 0.20	0.06	4.0
	Stride Length_LL (meters)	1.71 ± 0.18	1.76 ± 0.20	0.07	3.8
	Step Time (sec)	0.55 ± 0.06	0.53 ± 0.05	0.02	4.6
	Step Length (meters)	0.71 ± 0.10	0.73 ± 0.11	0.04	5.1
	Step Length_LL (meters)	0.85 ± 0.10	0.87 ± 0.12	0.04	5.1
	Step Width (meters)	0.19 ± 0.06	0.20 ± 0.05	0.02	11.3
FCWS					
	Gait Speed (m.s^−1^)	1.95 ± 0.33	1.97 ± 0.26	0.06	3.0
	Gait Speed_LL (m.s^−1^)	2.31 ± 0.37	
2.34 ± 0.29	0.07	2.8
	Cadence (steps/min)	134.9 ± 15.5	135.3 ± 13.5	2.51	1.9
	Stride Time (sec)	0.90 ± 0.10	0.89 ± 0.09	0.02	2.6
	Stance Time (sec)	0.58 ± 0.07	0.57 ± 0.07	0.02	3.7
	Double Leg Support Time 1 (sec)	0.13 ± 0.02	
0.13 ± 0.02	0.01	8.0
	Double Leg Support Time 2 (sec)	0.13 ± 0.02	
0.13 ± 0.02	0.01	7.7
	Single Leg Support Time (sec)	0.32 ± 0.04	
0.32 ± 0.03	0.01	3.8
	Swing Time (sec)	0.32 ± 0.04	0.32 ± 0.03	0.01	4.6
	Stride Length (meters)	1.68 ± 0.21	1.70 ± 0.18	0.06	3.3
	Stride Length_LL (meters)	2.01 ± 0.22	
2.04 ± 0.19	0.07	3.4
	Step Time (sec)	0.44 ± 0.05	0.44 ± 0.05	0.02	3.6
	Step Length (meters)	0.83 ± 0.12	0.85 ± 0.10	0.04	4.9
	Step Length_LL (meters)	1.0 ± 0.12	1.02 ± 0.11	0.05
	4.8
	Step Width (meters)	0.22 ± 0.07	0.23 ± 0.07	0.03	12.0

The absolute reliability of STP, reported here as SEM%, is comparable to previously reported clinical gait outcome measures (Flansbjer et al., 2005). Spatiotemporal parameters have been used widely in determining clinical outcomes and differences between healthy and pathological populations. Changes to joint RoM have been reported to influence spatiotemporal parameters; for example, in individuals with Parkinson’s disease, reduced step length, increased double support time, and cadence have been informative in determining the stage of the disease (Mirelman et al., 2019). Literature has reported differences in pathological gait compared to healthy individuals as 0.2–0.42 m for stride length, 0.05–0.13 m for step width, 0.06–0.14 m for step length (Chen et al., 2005a; Albani et al., 2014; Pistacchi et al., 2017; Bouça-Machado et al., 2020). Considering the data reported in the literature as expected differences, then we can proceed with confidence that gait analysis performed with Theia3D MLMC is sensitive and can discriminate spatiotemporal deviations for clinical use. Similar to kinematics, the measurement errors of spatiotemporal parameters reported in this study may help to avoid over-interpretation of results in studies employing Theia3D.

### Curve Analysis

The RMSD ranged from 0.96° to 3.56° for SSWS (see Figure 2) and 0.97° to 3.71° for FCWS (see Figure 3). Across all joints, planes of motion, and walking tasks, the lowest RMSD was observed in hip frontal plane angles (see Figures 2D, Figures 3D) while the highest RMSD was observed at the knee joint in the transverse plane (see 2H, 3H), across both walking tasks.

### SEM

#### Sagittal Plane

Our data revealed SEM values of less than 3° for sagittal plane kinematics for all three joints (range: 1.5°–2.84° for SSWS and 1.40°–2.83° during FCWS; see Tables s 3, 6).

#### Frontal Plane

Kinematics for all three joints produced errors ranging from 0.91° to 3.01° during SSWS and 0.91° to 3.25°during FCWS. For both walking tasks (see Tables s 4, 7), ankle RoM produced the highest SEM >3°. The lowest values were observed for hip joint maximum abduction angle during swing phase (SEM <1°) across both walking tasks.

#### Transverse Plane

For all joints, the largest measurement errors were observed in the transverse plane (range: 2.80°–4.95° in SSWS and 2.52°–5.50° for FCWS; see Tables s 5, 8). Ankle and knee RoM angles for both walking tasks produced measurement errors >5°. Hip joint kinematics produced the lowest SEM across both walking tasks.

#### Spatiotemporal Parameters

All spatiotemporal parameters produced SEM% ranging between 2.6 and 16.8 percent during SSWS and 1.9 and 12.0 percent during FCWS (see Table 9). During SSWS, first and second double limb support time, and stride width produced SEM% >10%. During FCWS, only stride width produced an SEM% above >10% (12.0%).

## Discussion

To our knowledge, this is the first study to report measurement errors associated with the Theia3D MLMC software. To date, no study has reported concurrent measurement of absolute reliability for both full curve and discrete point analysis kinematics and spatiotemporal parameters using either marker-based or markerless 3D motion capture. Our results show that MLMC is able to provide acceptable measurement error for the assessment of both full curve and discrete point kinematics, and spatiotemporal parameters. The full curve analysis reported here shows acceptable error for between-day kinematics examination. For between-day reliability, gait data acquired with MLMC largely produced measurement errors <3°. The highest measurement error was observed in the transverse plane, a typical observation when performing 3D motion capture of human walking (McGinley et al., 2009). This result is consistent with previous findings and not surprising as transverse plane motions are susceptible to error due to the limited range of movement (McGinley et al., 2009). Specifically, McGinley et al. (2009) identified hip rotation angles to be susceptible to the highest error when using the current gold standard, marker-based 3D motion capture. The results of this study, however, show that, with the exception of hip joint RoM during FCWS (3.39°), the hip transverse plane motion produced RMSD and SEM values <3° which tend lower than data acquired with marker-based motion capture (McGinley et al., 2009). The SEM of spatiotemporal parameters reported here is similar to previous studies that have examined reliability using 3D motion capture, IMU sensors, or pressure sensing walkways (Paterson et al., 2008; Meldrum et al., 2014; Posada-Ordax et al., 2021).

The low measurement errors observed from data acquired with Theia3D MLMC reported in this study demonstrate the sensitivity necessary to detect small changes in clinically important outcome measures. Thus, Theia3D provides confidence for detecting gait pathology or monitoring outcomes in response to rehabilitation. Data currently reported in the literature, derived from marker-based motion capture, reveal mean differences greater than the SEM values reported here. For example, Albani et al. (2014) examined sagittal plane kinematic differences between healthy controls and individuals with Parkinson’s disease. The authors reported differences between healthy and patient groups of 5.1° for hip RoM, 7.5° for knee RoM, 1.7° for max knee flexion angle during swing, and 1.3° for ankle RoM. Chen et al. (2005a) reported a difference of 20.8° between stroke survivors and healthy controls for sagittal plane max knee angle during swing. While an SEM value of 2.47° for the ankle sagittal plane RoM is reported here, Albani et al. (2014) reported a difference of 1.3° between healthy and impaired gait. However, within Parkinson’s disease, ankle kinematics are less likely to be altered when compared to healthy (Zanardi et al., 2021). Regardless, the knowledge of SEM values of hip, knee, and ankle kinematics should aid future studies using Theia3D and avoid potential over-interpretation of results when reporting kinematic differences.

Full curve examination affords the ability to detect possible deviations that may not be detected using discrete analysis. While values at a defined point may be similar between sessions, such evaluation does not mean that the pattern of motion was consistent across the full gait cycle. Our inter-session curve analysis reveals low magnitude differences for the entire gait cycle providing further confidence for both biomechanists and clinicians who desire to use MLMC for kinematic investigation.

Previous examinations of the reliability and validity of MLMC, Theia3D specifically, have been performed by the developers who bring extensive knowledge and experience with both deep learning (AI) and biomechanical analysis (Kanko et al., 2021a, b). The current study has several differences from the experiments conducted by Kanko and colleagues. Kanko et al. (2021b) established reliability using data collected in a dedicated laboratory setting, from eight college-aged participants, while controlling for clothing and footwear. In the present study, we leveraged the ability to perform 3D gait analysis outside of a laboratory, working instead in accessible environments such as community centers and clinical facilities representative of where this new technology may be used to evaluate clinical populations and functional outcomes. Additionally, we sampled a diverse group of individuals and explicitly did not control for their attire. We sought to determine how realistic it will be to use this tool for the evaluation of gait dysfunction in clinical populations, particularly individuals participating in neurorehabilitation. Our data show strong evidence of low measurement error, even in what may be traditionally considered non-ideal conditions for 3D motion analysis, demonstrating strong potential for use of MLMC in clinical environments.

While representing significant and important advances in biomechanical technologies, it is important to note that, similar to marker-based motion capture tools, currently available commercial MLMC products are not turnkey systems. Prior to conducting the present study, our team performed extensive development work (described in the companion article, McGuirk et al., 2022) to determine the requisites (e.g., location, optimal capture volume, camera configuration, lighting, collection time, sampling frequency, and the number of trials required for each task and location) for feasibly acquiring 3D kinematics in free living spaces. This development process revealed the majority of clothing allows for detection of expected limb and segment motion. One challenge encountered, however, was with calf/ankle length skirts, which did not allow the modeling of the pelvis and legs. Of the 21 individuals who participated in this study, 10 wore similar clothing for both sessions (e.g., t-shirt and shorts). Six individuals wore shorts or a short (above-knee length) skirt for one session and switched to long trousers for the other; all six also wore different shoes. One individual switched from sandals to shoes between sessions; another walked barefoot for both sessions.

## Conclusion

The results of this study show that 3D gait analysis conducted in open, participant-facing environments with markerless motion capture affords repeatable, accurate 3D gait data suitable for both biomechanists and clinicians. Measurement error of 3D kinematics examined through both full curve and discrete point analysis and spatiotemporal parameters was low, providing a basis for interpretation of both clinical status and monitoring rehabilitation outcomes. This study shows that implementation of Theia3D MLMC does not require specific procedures to control for clothing as is the case for marker-based motion capture. The findings of this study also support the feasibility of using the MLMC tools in various settings and populations. While the reliability of the data acquired using markerless motion capture was comparable to that reported from marker-based systems, in some cases—such as hip transverse plane kinematics—we observed lower measurement errors. Together, these findings provide confidence in MLMC and indicating strong potential for its future use in the neurorehabilitation setting.

## Data Availability Statement

The raw data supporting the conclusions of this article will be made available by the authors, without undue reservation.

## Ethics Statement

The studies involving human participants were reviewed and approved by University of California, Davis Institutional Review Board. The patients/participants provided their written informed consent to participate in this study.

## Author Contributions

CP and TM conceived and designed the study. All authors contributed to data collection. TM and WS processed, SR analyzed, and SR and CP interpreted, the data. EP developed electronic data collection tools. All authors contributed to writing the manuscript and approved the submitted version.

## Conflict of Interest

The authors declare that the research was conducted in the absence of any commercial or financial relationships that could be construed as a potential conflict of interest.

## Publisher’s Note

All claims expressed in this article are solely those of the authors and do not necessarily represent those of their affiliated organizations, or those of the publisher, the editors and the reviewers. Any product that may be evaluated in this article, or claim that may be made by its manufacturer, is not guaranteed or endorsed by the publisher.
